# Diagnosis of Indigenous Non-Malarial Vector-Borne Infections from Malaria Negative Samples from Community and Rural Hospital Surveillance in Dhalai District, Tripura, North-East India

**DOI:** 10.3390/diagnostics12020362

**Published:** 2022-02-01

**Authors:** Ipsita Pal Bhowmick, Apoorva Pandey, Sarala K. Subbarao, Rocky Pebam, Tapan Majumder, Aatreyee Nath, Diptarup Nandi, Analabha Basu, Apurba Sarkar, Saikat Majumder, Jotish Debbarma, Dipanjan Dasgupta, Arup Borgohain, Rajdeep Chanda, Mandakini Das, Karuna Gogoi, Kongkona Gogoi, Pyare Laal Joshi, Harpreet Kaur, Biswajyoti Borkakoti, Dibya Ranjan Bhattacharya, Abdul Mamood Khan, Satyajit Sen, Kanwar Narain

**Affiliations:** 1 Regional Medical Research Center-Northeast Region (RMRC-NE)-ICMR, Dibrugarh 786001, India; jd45683968@gmail.com (J.D.); dipanjandasgupta89@gmail.com (D.D.); mandakini.mail@gmail.com (M.D.); karunarmrc@gmail.com (K.G.); koko1.gogoi@gmail.com (K.G.); biswaborkakoty@gmail.com (B.B.); drbhattacharyya@yahoo.com (D.R.B.); kanwar_narain@hotmail.com (K.N.); 2 Indian Council of Medical Research (ICMR), Ramalingaswami Bhavan, New Delhi 110029, India; apoorva.icmr@gmail.com (A.P.); kaurh.hq@icmr.gov.in (H.K.); amkhan.hq@icmr.gov.in (A.M.K.); 3 Formerly National Institute of Malaria Research, Indian Council of Medical Research ICMR, New Delhi 110029, India; subbaraosk@gmail.com; 4 North Eastern Space Applications Centre, Department of Space, Government of India Umiam, Umiam 793103, India; rocky.pebam@gmail.com (R.P.); aatreyeen04@gmail.com (A.N.); arupborgohain@gmail.com (A.B.); 5 Department of Microbiology, Agartala Government Medical College, Agartala 799006, India; drtapan1960@gmail.com (T.M.); apurbasarkar020@gmail.com (A.S.); majumder.saikat@gmail.com (S.M.); 6 National Institute of Biomedical Genomics, Kalyani 741251, India; dn1@nibmg.ac.in (D.N.); ab1@nibmg.ac.in (A.B.); 7 Department of Forestry, Mizoram University, Aizawl 796004, India; rajdeepchanda1991@gmail.com; 8 Roche Diagnostics India Pvt. Ltd., Mumbai 400069, India; 9 Formerly National Vector Borne Disease Control Program (NVBDCP), New Delhi 110054, India; doctorjoshi00@gmail.com; 10 Regional Office of Health and Family Welfare, Kolkata 700106, India; rohfw.kolkata1@gmail.com

**Keywords:** acute febrile illness, infectious diseases, malaria, non-malaria vector-borne diseases, Dengue, Chikungunya, Japanese encephalitis, scrub typhus, leptospirosis, malaria-endemic region, community fever surveillance

## Abstract

The aetiology of non-malaria vector-borne diseases in malaria-endemic, forested, rural, and tribal-dominated areas of Dhalai, Tripura, in north-east India, was studied for the first time in the samples collected from malaria Rapid Diagnostic Kit negative febrile patients by door-to-door visits in the villages and primary health centres. Two hundred and sixty serum samples were tested for the Dengue NS1 antigen and the IgM antibodies of Dengue, Chikungunya, Scrub Typhus (ST), and Japanese Encephalitis (JE) during April 2019–March 2020. Fifteen Dengue, six JE, twelve Chikungunya, nine ST and three Leptospirosis, and mixed infections of three JE + Chikungunya, four Dengue + Chikungunya, three Dengue + JE + Chikungunya, one Dengue + Chikungunya + ST, and one Dengue + ST were found positive by IgM ELISA tests, and four for the Dengue NS1 antigen, all without any travel history. True prevalence values estimated for infections detected by Dengue IgM were 0.134 (95% CI: 0.08–0.2), Chikungunya were 0.084 (95% CI: 0.05–0.13), Scrub were 0.043 (95% CI: 0.01–0.09), and Japanese Encephalitis were 0.045 (95% CI: 0.02–0.09). Dengue and Chikungunya were associated significantly more with a younger age. There was a lack of a defined set of symptoms for any of the Dengue, Chikungunya, JE or ST infections, as indicated by the k-modes cluster analysis. Interestingly, most of these symptoms have an overlapping set with malaria; thereby, it becomes imperative that malaria and these non-malaria vector-borne disease diagnoses are made in a coordinated manner. Findings from this study call for advances in routine diagnostic procedures and the development of a protocol that can accommodate, currently, in practicing the rapid diagnosis of malaria and other vector-borne diseases, which is doable even in the resource-poor settings of rural hospitals and during community fever surveillance.

## 1. Introduction

Infectious diseases are a significant public health burden in India, causing morbidity and mortality [1], with fever being the most common clinical symptom. Acute Febrile Illness (AFI) is defined as any illness associated with a rapid onset of fever of 2 weeks or a shorter duration, caused by diverse pathogens, but without the evidence of organ or system-specific aetiology [2]. Malaria is one of those diseases that cause fever and is endemic in many parts of developing countries, including India. These areas are often rural, especially in north-east (NE) India, and include tribal populations living in forest-fringes, where forest-dwelling disease-carrying mosquito vectors are common [3].

The availability of sensitive Rapid Diagnostic Kits (RDTs) for malaria diagnosis, either by the regular active door-to-door community-level fever surveillance, at village health camps, or by passive surveillance at the health care facilities serving several villages, has allowed population-level malaria testing in endemic areas. However, once malaria is ruled out, identifying the underlying cause of febrile illness becomes challenging [4]. The importance of non-malaria fevers (NMFs) is severely undermined due to under-diagnosis, especially in peri/semi-urban or rural areas, where diagnostic facilities are not available [5,6,7]. Several non-malarial vector-borne diseases (NMVBDs) have no point-of-care detection facility because diagnostic tests against these pathogens are expensive and require skilled personnel and testing platforms. It is also difficult to distinguish between different causes of febrile illnesses clinically due to overlapping clinical manifestations [8]. The establishment of the nationwide network of Viral Diagnostic Research Laboratories (VRDLs) by the Department of Health Research and the Indian Council of Medical Research (ICMR), Government of India [9], has led to significantly improved diagnosis for NMF illnesses. However, VRDLs mainly cater to severe referral AFIs, samples from outbreak investigations and patients from respective city catchment areas. 

Previous studies in Asia have identified the causes of several NMF illnesses [10,11,12], including some from India [2,4,8,9,13,14,15,16,17,18,19,20], but few from NE India [21,22,23], with none specifically from malaria-endemic regions. These studies in India are either conducted after outbreaks or are hospital-based [2,18,21,22,24], and still do not represent community-level disease burden. Frequent outbreaks of Acute Encephalitis Syndrome (AES) have been reported in different parts of India. Recently, Scrub Typhus (ST) has been considered a significant cause of AES in Gorakhpur in Uttar Pradesh, Bihar, Assam [14,19].

In this context, it is crucial to study the aetiology of NMFs in the malaria-endemic regions, and conduct both rural hospital and community-based studies. Several tribal-dominated forested pockets of the Dhalai District, Tripura in NE India, fall under the malaria-endemic area [25], with malaria outbreak histories recently in 2014 [3] and 2018. The present study was designed to determine the aetiology of acute NMFs during one year of active community-level surveillance in remote forested villages, and admitted febrile cases in a rural primary hospital of Dhalai along with demographical, ecological and meteorological attributes. The findings call for integrated fever surveillance, i.e., current malaria surveillance encompassing these NMVBDs, which is important for doing proper case management, thereby reducing morbidity, and formulating vector control strategies to prevent outbreaks of these diseases from these kinds of areas in future.

## 2. Materials and Methods

### 2.1. Study Area

The Dhalai district lies in the north-east part of Tripura. The study was conducted on the admitted febrile patients at the Ambassa Primary Health Centre (PHC), which serves the small township area of Ambassa Municipality, Village councils and Autonomous District Council (ADC) villages of tribal people. In addition, community/village surveillance was carried out in the malaria-endemic villages of two Sub Centres (SCs), Gurudhanpara and Shikaribari, in the ADC area, with an average Annual Parasite Incidence (API) of >200 and ~50, respectively. A few samples were also collected from villages under adjacent SCs under Ganganagar PHC.

### 2.2. Sample Collection

The venous blood samples were collected by project technicians from NMF patients from April 2019 to March 2020.

#### 2.2.1. Records of Fever and Malaria Cases

**Figure 1:**
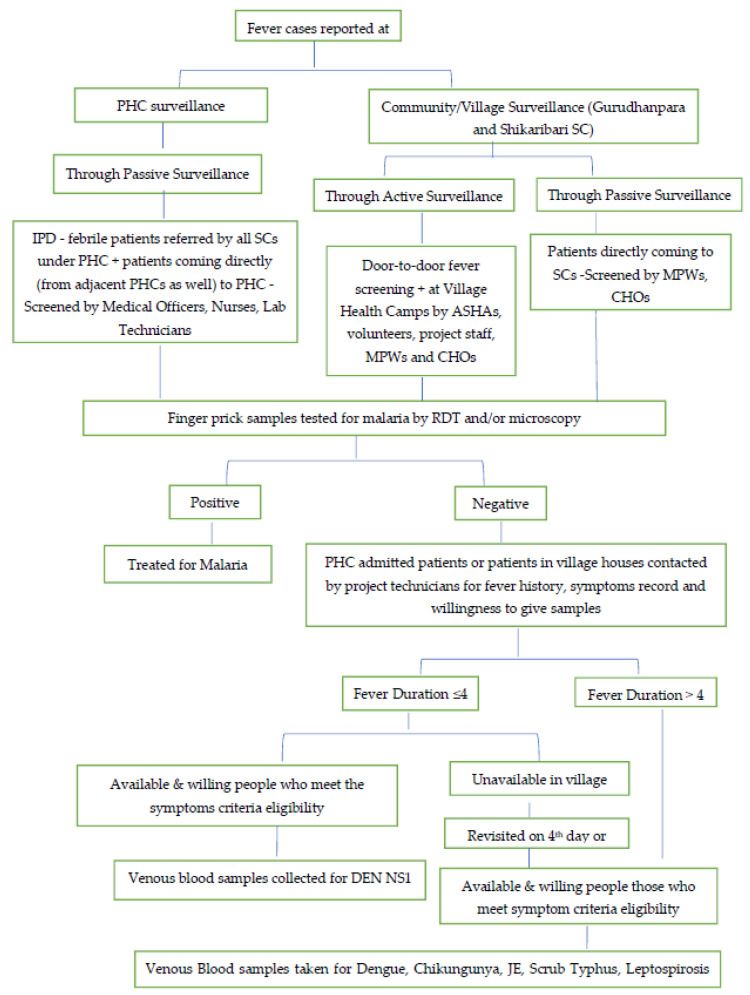
Flow chart of sample collection from Non-Malarial Febrile patients admitted in PHC and those identified by community/village surveillance: All febrile cases were diagnosed for malaria using RDT by ASHAs, village volunteers, project technicians at village household-based surveys, and MPWs or CHOs at SCs or during field-visits, and by microscopy/RDT by Laboratory Technicians at PHC OPD or Nurses at IPD. Malaria-negative patients at PHC IPD or village households were contacted for venous blood sample collection, as described in the flow chart, by the project lab technicians of ICMR-NE RMRC Dibrugarh. ASHA = Accredited Social Health Activists, RDT = Rapid Diagnostic Kit, MPW = Multipurpose workers, CHO = Community health Officers, OPD = Out Patient Department, IPD = Inpatient Department.

Patients’ names were compiled from respective fever registers by manually checking for duplication. Malaria-negative febrile patients at PHC Inpatient Department (IPD) or village households were contacted for sample collection as described in detail in Figure 1 Flowchart.

#### 2.2.2. Exclusion Criteria

Less than 1-year-olds, febrile patients whose symptoms did not match the criteria (Table S1), and those determined to be unfit for inclusion by a physician or unwilling were excluded.

#### 2.2.3. Village and PHC Collections

Malarial RDT negative patients, 192 from the community survey, 64 from Ambassa PHC (61 from IPD, three from OPD), and four from IPD of District Hospital, Kulai, were screened for NMVBDs.

#### 2.2.4. Medical History Records

Fever duration and symptoms were recorded as per predefined VRDL Tripura proforma based on Integrated Disease Surveillance Program (IDSP), Tripura guidelines (Table S1). The patients were asked about these symptoms in their local language with the help of village facilitators. 

### 2.3. Sample Processing

The serum was separated by centrifugation at Ambassa PHC and stored at −20 °C for later use or sent for testing to VRDL, Agartala, immediately, depending on the transport feasibility. We tested for Dengue, Chikungunya, Japanese Encephalitis (JE) IgM antibodies by ELISA (NIV IgM Capture ELISA kits), and Dengue NS1 antigen and ST (InBios Dengue NS1 ELISA and ST Detect^TM^ IgM/IgG Kits) as per manufacturer’s instructions. In addition, we tested a small subset, irrespective of symptoms for Leptospirosis, using the PanBio Leptospira IgM ELISA kit at VRDL Dibrugarh, Assam. A subset of samples was also tested outside the recommended symptoms and duration criteria of the IDSP guidelines (Tables S1 and S2). A small subset of sample verification at the ICMR-National Institute of Virology, Pune, was done as per the protocol. Informed consent was obtained from the patients. 

### 2.4. Demographic Census

We conducted a geo-referenced census in 18 study villages during February–April 2019. Information on age, occupation, long-lasting insecticidal nets (LLINs), possession and usage, livestock, and house wall and roof status were recorded.

### 2.5. Meteorological Data Collection

The study used spatial and 30-min temporal resolution rainfall data from NASA’s GPM project, and temperature and relative humidity data from the MERRA-2 model. The daily maximum, minimum, average temperature, NDVI and relative humidity on a spatial resolution of 50 km × 62.5 km were plotted from the datasets downloaded in NetCDF formats and extracted using software codes.

### 2.6. Preparation of Ecological Maps

Land use land cover (LULC) mapping was prepared using the orthorectified Indian Remote Sensing satellite data, Cartosat-1 (2.5 m) and LISS-IV (5.8 m), employing on-screen visual interpretation techniques in Geographical Information System (GIS) platform. Major LULC categories, subcategories were delineated and updated using the latest data (2019) on the spatial layer, initially prepared under NRSC/ISRO’s Space-based Information Support at 1:10,000 scales. In addition, field verifications were made by the project team to check for the accuracy of the interpreted data. 

### 2.7. Data Analysis

The symptoms, clinical findings, demographic data and laboratory results were used for descriptive analysis in IBM SPSS Statistics 20. The Chi-square test was used to assess the differences between different age groups in Epi. Yates’ Continuity correction test was applied to chi-square in R version 4.1.0 software environment for statistical computing. As Dengue was tested by both IgM and NS1, these tests were considered separately for calculation. IBM SPSS Statistics 20 and Epi InfoTM version 7, CDC, USA, was used. For calculation of true prevalence values, Epitools, an epidemiological calculator, was used [26]. All the infections, including the single and mixed infections were considered for each disease. Blaker’s confidence limits were used [27]. 

k-Modes clustering was used to test whether the four NMVBDs (JE, ST, Chikungunya, Dengue) had distinct symptoms. A set of nine symptoms (retro-orbital pain, myalgia, arthralgia, neck rigidity, altered sensorium, chills, irritability, headache, rash) were used for this analysis, and only cases which tested positive for a single infection were considered (*n* = 46). Leptospirosis was not considered because of the small numbers tested. A k of four was chosen, corresponding to the four diseases. The analysis was carried out using the klaR package in R version 4.1.0 software environment for statistical computing [28,29].

## 3. Results

### 3.1. Fever and Malaria Infections

The records of fevers and malaria infections in Ambassa PHC from April 2019 to March 2020, and Gurudhanpara and Shikaribari sub-centres (SCs) are given in Table 1 and Table 2, respectively. These data show that many fever cases are reported annually, with a high number of fever cases being reported to ASHAs, health volunteers, or SC MPWs. A high number of malaria cases are reported with the malaria Annual Parasite Incidence (API) for the study year, being ~10 for Ambassa PHC (malaria cases: 589, population ~61,000) and 122 for Gurudhanpara SC (malaria cases: 233, population ~1900) and ~57 for Shikaribari SC (malaria cases: 102, population ~1800). It is evident that a significant portion of the fever cases was non-malarial.

### 3.2. Vector-Borne Non-Malarial Fevers

**Figure 2:**
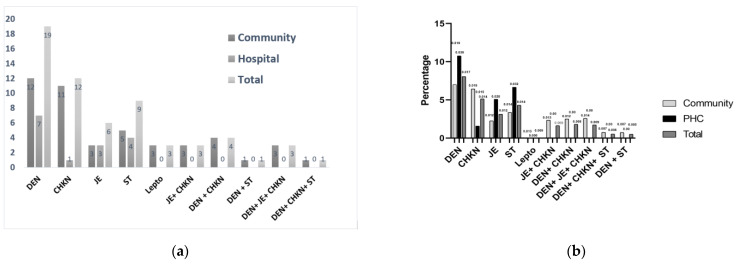
No. of positive cases and percent positivity of DEN, CHKN, JE, ST, Lepto, and mixed infections. (**a**) Higher no. of cases has been detected from the surveillances in communities/villages than from Hospital /PHC. Leptospirosis and all mixed infections have been detected only in community-based surveillance. (**b**) Higher positivity rate was found in community/village surveillance for CHKN and vice versa for DEN, JE and ST. DEN = Dengue, CHKN = Chikungunya, JE = Japanese Encephalitis, ST = Scrub Typhus, Lepto = Leptospirosis.

Out of 260 malaria negative febrile samples collected (females—141; males—119), 61 were found positive—nineteen Dengue, twelve Chikungunya, six JE, nine ST and three Leptospirosis, and mixed-infections, viz., three JE + Chikungunya, four Dengue + Chikungunya, three Dengue + JE + Chikungunya, one Dengue + Chikungunya + ST and one Dengue + ST. The details of positive infections found from PHC and villages, and test positivity rates are shown in Figure 2a,b. Except for one case, none of these cases were found to have any travel history outside their home area to any known endemic zone for NMVBDs. Two patients from the same family were positive for both JE and Chikungunya IgMs in May 2019 and again in January 2020 for JE, Chikungunya and Dengue IgMs. As the gap between these two sets of infections was more than six months, and patients were febrile with several different symptoms the second time, January infections were considered separate infections. For true prevalence estimations of single, as well as mixed infections, were considered for each disease. True prevalence values estimated for infections detected by Dengue IgM were 0.134 (95% CI: 0.08–0.2), Chikungunya 0.084 (95% CI: 0.05–0.13), Scrub 0.043 (95% CI: 0.01–0.09), Japanese Encephalitis 0.045 (95% CI: 0.02–0.09). The details are given in the Table S3.

### 3.3. Percentage Prevalence of the Symptoms for Each Disease as Reported by the Patients Clustering Analysis between the Symptoms

Headaches and body ache were found most commonly in all infections, followed by Myalgia in Dengue and Chikungunya (Figure S1). Chills were reported in many infections of Dengue and JE. Irritability was more common in JE. Typical for Dengue and Chikungunya, a rash was not reported in those infections, but a small fraction reported it in JE and ST infections. Retro-orbital pain, dark urine, abdominal pain and vomiting occurred in some patients in all five infections. Breathlessness was high from JE patients, while altered sensorium and a mental status change were seen from a few, including JE patients. Mixed infections have not been considered for symptom calculations; hence, the sample number is low for ST and Leptospirosis. Notably, eschar was not reported by any ST positive infection case. 

The cluster analysis is shown in Table S4. As can be seen, each of the four clusters contain multiple disease cases, instead of a single disease. It demonstrates a high degree of overlap between the nine symptoms for the four diseases. None of the four clusters corresponded to any single disease, implying a lack of a defined set of symptoms for any of the four diseases.

### 3.4. Diseases Tested Outside Criteria

A subset of samples tested outside the duration criteria of >4 days of fever for IgM or ≤4 days for NS1 antigen tests were found positive for NMVBDs (Table S2). In addition, a subset of cases whose symptom criteria lay outside the stipulated ones (Table S1) tested positive for JE (Table S5). 

### 3.5. Association with Age

Chi-square analysis showed the <15-year group had a significantly higher risk for Dengue and Chikungunya. After applying Yates’ continuity correction, the higher risk in <15-year age group in Dengue remained significant. There was no significant difference or trend in JE or ST. 

**Figure 3:**
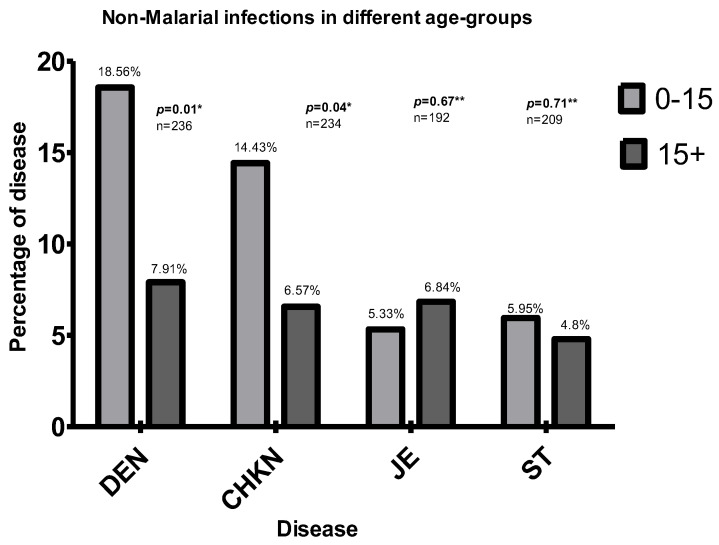
Age group-wise disease positivity percentage of different diseases including mixed infections. It shows significantly more risk of DEN in the lower age group. Both DEN and CHKN show higher risk in the older age group when mixed infections are considered. DEN = Dengue, CHKN = Chikungunya, JE = Japanese Encephalitis, ST = Scrub Typhus. * Statistically significant; ** Statistically not significant.

For JE and ST, there was no significant difference between different age groups (Figure 3). Higher proportions of JE, ST and mixed infections involving them were in the >15 age group, but without statistical significance (Figure 3).

### 3.6. Demographic Pattern and Vector Control Practices of All Tested and Positive Cases from the Villages

Analysed from the village census data (Table 3), the majority of the febrile and NMVBD positive people were found to have the habit of always sleeping under the new LLINs (Table S6). *Jhum* cultivation, where villagers slash and burn the forests in one hillock deep inside the forest area and cultivate for a year, staying in temporary *Jhum* huts or going up and down from villages regularly through dense forest-areas, was the main occupation of the febrile villagers tested for NMVBDs. No pattern for houses with different types of walls and roofs or livestock possession was found among the disease positives.

### 3.7. Ecological and Meteorological Correlates

**Figure 4:**
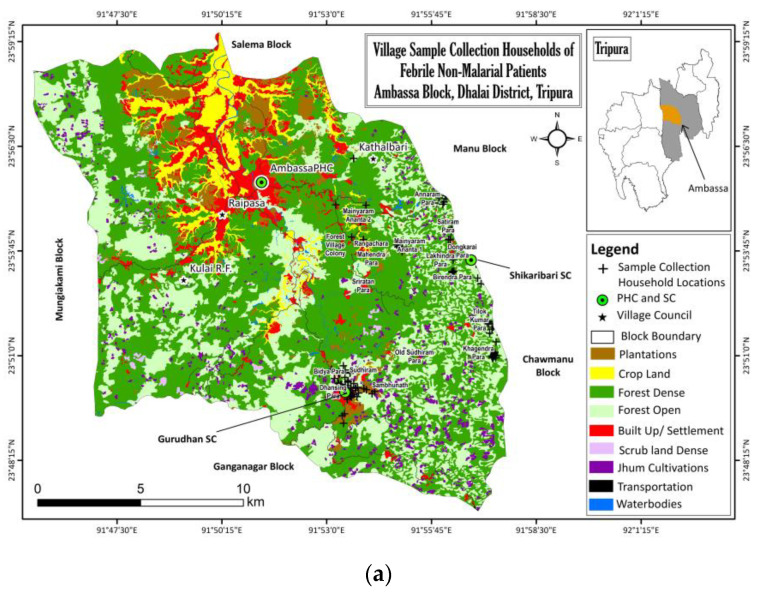

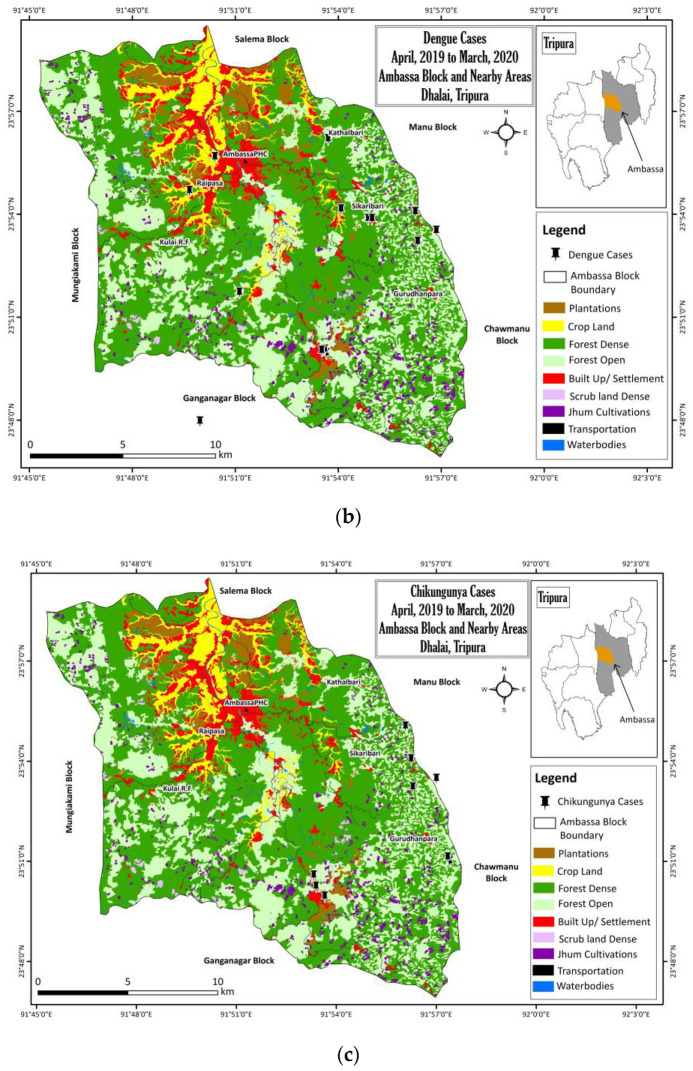

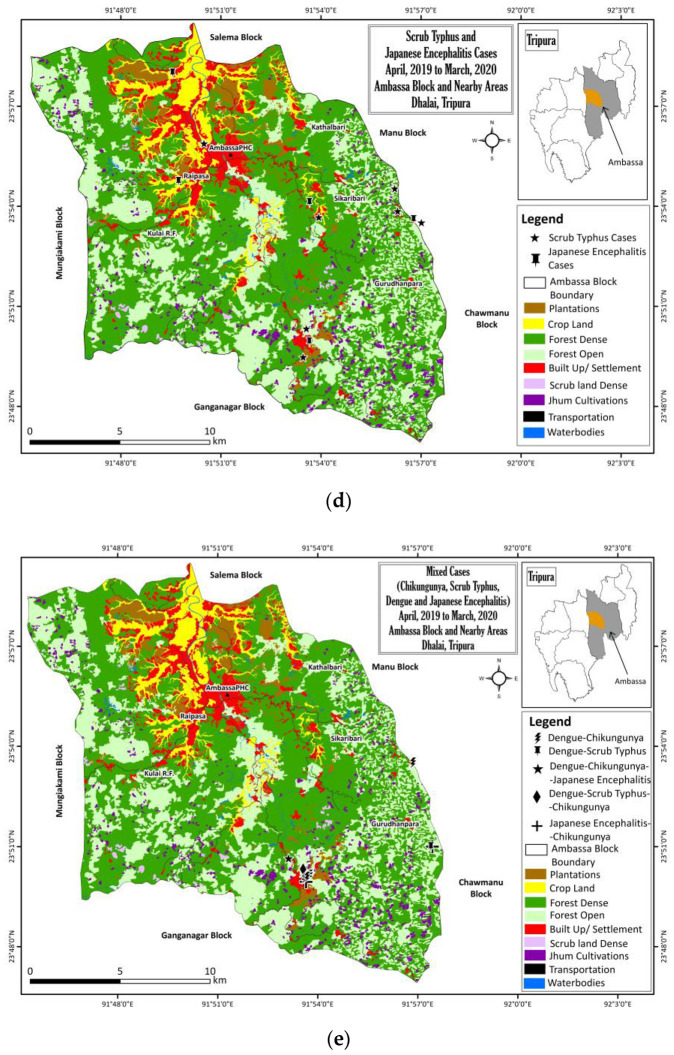
Land Use Land Cover (LULC) Maps of Ambassa Block. LULC mapping was prepared using the orthorectified Indian Remote Sensing satellite data, Cartosat-1 (2.5 m) and LISS-IV (5.8 m), employing on-screen visual interpretation techniques in the GIS platform. Field verifications were made by the project team to check for the accuracy of the interpreted data. Geolocations of village sample collection and NMVBD positive households were collected from the census and plotted on the map for analyses of the proximity of the households to the *Jhum* fields/plantations/forests, etc. Positive cases found from the PHC IPD collection were plotted for their village address. Geolocations of (**a**) all sample collection households from villages, (**b**) Dengue positive households, (**c**) Chikungunya households, (**d**) Japanese encephalitis and Scrub Typhus households, and (**e**) mixed infection case household locations from village and PHC based surveillance. Maps show the villages located mainly inside or nearby deep forested area with *Jhum* cultivation.

The ecological maps of Ambassa block plot the distribution of households sampled from the villages (Figure 4a) as Figure 4b–f show the location of febrile, and most of the positive infection houses, situated in the deep-forested regions. Samples collected from PHC could not be mapped to their household geolocation accurately, but plotted to the address provided. It showed some infections in small township areas, but agricultural lands, rubber plantations, etc., mostly surround those areas.

**Figure 5:**
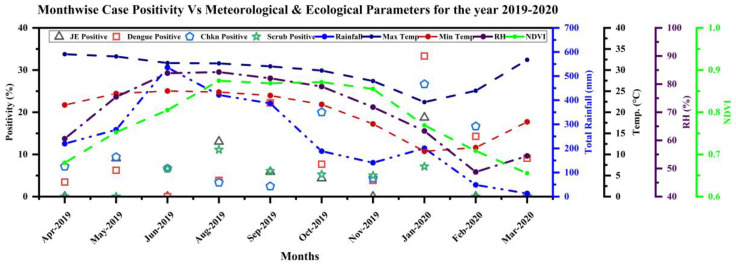
Variation in monthly meteorological parameters during the study period. The temperature and relative humidity data were taken from NASA’s MERRA-2 model. The used products are the daily maximum (T2MMAX), minimum (T2MMIN), average temperature (T2MMEAN), and relative humidity. Month-wise sample positivity for Dengue, Chikungunya, JE, Scrub Typhus diseases were plotted on the X-axis to examine the relationship with the meteorological parameters at the time cases were identified. The yearlong transmission for all the diseases with relatively high positivity rate in dry winter months was seen. MERRA = Modern-Era Retrospective analysis for Research and Applications, JE = Japanese Encephalitis.

NMVBDs occurred throughout the year, with significant positivity even in the winter months (Figure 5). For the mixed infections, the possibility of getting some infection a few months back cannot be ruled out as IgM can persist for months. Hence, all the individual infections detected in a mixed-infection set may not be responsible for causing the current febrile case. Therefore, only single-disease infections were used for these plots. The infections have been segregated month-wise depending on the date of sample collection.

In some cases, fever duration at the time of collection was >5 days. Hence, samples collected at the beginning of a month can be the infection of the previous month. Taking together the incubation time in humans, the cycle time in mosquitoes and the time taken from breeding to mosquito maturation, it would be better to interpret the monthly data correlation with meteorological data of the previous month.

## 4. Discussion

To the best of our knowledge, this is the first study to diagnose NMF cases collected from the community door-to-door fever surveillance in remote malaria-endemic rural forested regions of India, from the patients termed as malaria negative by the routine surveillance system. It thus points towards gaps in the diagnosis system in malaria-endemic areas and adds a new dimension to the growing knowledge of aetiologies of NMFs in India. Malaria has been the only priority for fever surveillance in these villages, located in a malaria-endemic zone, where the presence of other vector-borne diseases has never been attempted to be diagnosed at the community level. They have been neglected even in the rural PHC level routine surveillance and research studies as well.

We have shown that this area is highly malarious, where a substantial number of malaria cases can be seen with an API of ~10 for Ambassa PHC in the study period, and an API of ~122 for Gurdhanpara SC, and ~57 for Shakaribari (Table 2).

We demonstrated here a high burden of fever cases in these malaria-endemic areas. Approximately 18,000 fever cases in the PHC (Ambassa) of a ~60,000 population (Table 1), and 2500 in the two SCs (Gurudhanpara and Shikaribari) of a total ~4000 population (Table 2) were reported throughout the year. These cases were reported via passive surveillance at the PHC and SCs, or by active surveillance in the community. A major fraction of febrile people from these remote villages rely upon active surveillance, and the rest go to SCs (Table 2). Very few febrile people from the remote ADC villages go directly to PHCs unless referred from SCs, and even if referred, not all go to the PHCs. Reasons are manifold, such as faraway locations, engagement in *Jhum* in deep forests (Figure 4), poor availability of transport, transportation cost, loss of daily work for the accompanying persons and a general unwillingness among the villagers to visit PHCs except for disease severity. Hence, only PHC based studies for NMVBDs miss many fever cases, which we tried to address through the inclusion of community surveillance in this study. 

For the PHC febrile cases, NMFs remain undiagnosed, and mostly symptomatic management is done for NMFs unless referred to tertiary care hospitals at the State Capital. There is no aetiological study published so far for rural PHC based NMFs in Tripura. Even in these highly malaria-endemic areas, malaria accounted for only 10–15% of febrile cases. Hence, this study attempted to shed light on the aetiology of NMFs in the PHC admitted cases, also.

We detected JE, ST, Leptospirosis, and even Dengue and Chikungunya, and mixed-infections mainly termed urban, semi-urban, or semi-rural, in these forested rural areas (Figure 2a). The absence of any travel history indicates local transmission of NMVBDs, warranting a deeper look, particularly with many NMFs reported at the PHC (Table 1) or from community surveillance (Table 2). A positivity rate of 2–8% (Figure 2b) indicates high transmission of the diseases. Therefore, we can expect many NMVBDs if their surveillance is integrated with routine surveillance programmes in these areas. Although the number of febrile patients screened is not high, considerably high positivity values confirmed the occurrence and intensive transmission of NMVBDs. This study indicates that many cases are missed as they are never supposed to be tested for these diseases in the current routine diagnostic protocol in these rural hospitals or community settings. Samples tested by ELISA in VRDL at Agartala Government Medical College (AGMC), Tripura, in 2017 and 2018 showed that out of total 4530, 7138 and 11,448 fever samples tested, 15.1%, 12.4% and 12.1% samples were positives for JE, Dengue and Chikungunya, respectively (Unpublished data, VRDL, Tripura). As these samples were mainly for the patients directly admitted at the tertiary care hospitals, such as AGMC or Tripura Medical College, which mainly include the catchment township area cases and many with travel histories, or the referred severe febrile cases from District Hospitals or Primary Health Centres, the inclusion of the indigenous samples from remote, rural areas, especially the febrile patients from the community surveillance can increase the numbers of NMVBDs a lot, as our findings suggest. 

Age group analysis showed a preponderance of Dengue and Chikungunya in the <15 age group (Figure 3), indicating possible hyper-endemicity of Dengue and Chikungunya in the area. In hyper-endemic regions of Asia, Dengue was shown to affect mainly children <15, while in America, these syndromes were found in all age groups [30]. Earlier, pan India studies on Dengue and Chikungunya reported very low seroprevalence in NE India [9,15]; however, this study indicates the possibility of some regions like the study area being endemic. Malaria-endemic regions can very well be endemic for NMVBDs, also. 

As this is the first report of these NMVB diseases from this area, there is no baseline information. Hence, the prevalence numbers estimated should be taken with caution, especially for community-based screening. A well-designed study is thus required to cover such areas in Tripura State and the whole of NE India to estimate the actual disease burden, comprising of the determination of the prevalence and seroprevalence for these diseases and other pathogens.

Our study also reported several mixed infections. However, we cannot completely rule out the chances of cross-reactivity. Furthermore, the mixed infection cases can be co-infections at the time of collection, or some can be from previous months, as IgMs have been shown to persist for months. Even in the case of co-infections, the causative agent(s) for the fever and other symptoms need to be nailed down. The present study, with its objectives, could not differentiate between these possibilities and requires the designing of further studies. If the screening of NMVBDs comes under the routine diagnostic protocol, it would be easier to resolve the mixed-infections related issues.

Leptospirosis was positive in three samples out of 51 tested from the villages, demonstrating the occurrence of Leptospirosis, though screening of significant numbers is required. Our findings also suggest the need for widening the symptoms and duration eligibility criteria as the tribal patients in these areas may not be very accurate in their presentation of various symptoms and fever duration, especially in the community surveillance by grass-root health workers. There was a lack of a defined set of symptoms for any of the Dengue, Chikungunya, JE or ST infections, as indicated by the k-modes cluster analysis. Interestingly, most of these symptoms have an overlapping set with malaria. Therefore, it becomes imperative that malaria and non-malaria vector-borne diseases’ diagnoses are made in a coordinated manner. 

The reluctance of the febrile patients to give venous blood was faced as many refused to give blood for testing these diseases, which are unknown to them, unlike malaria. Thus, it would be beneficial if routine blood collection for diagnosing malaria and NMVBDs from febrile patients was performed and awareness generated. 

Two major takeaways of this study are: (i) A routine diagnostic surveillance strategy encompassing NMVBDs along with malaria is required at PHC IPD and OPD, and community levels through the regular collection of febrile samples. Advancements are required to get the point of care diagnostics for NMVBDs with malaria. An integrated fever surveillance and management system are required at the grassroots level; (ii) Exclusive PHC based studies leave a substantial vulnerable population. Hence community-level studies are essential to ascertain the prevalence of NMVBDs and estimate the actual burden. This will be significant in estimating disease burden, preventing any disease outbreak by appropriate control measures and/or treatment, resulting in a decline in disease morbidity and mortality. It is worth mentioning that this study’s findings have prompted some surveillance at the rural health centre and community levels, especially for Dengue, with a rapid kit. Facilities for ELISA testings have recently started in some District hospitals. It helped report a very recent Dengue outbreak in some rural areas of Tripura (unpublished data).

With the presence of ST and Leptospirosis infections, a presumptive treatment regimen of Doxycycline for adults and Azithromycin for children and pregnant women can be thought of until the investigation reports come, in the case of malaria negative fever patients, to save them from morbidity and mortality. A study of such treatment for children presented with fever in peripheral health facilities in Gorakhpur, Uttar Pradesh, had shown promising efficacy in preventing AFI progression to AES [31]. Immunisation with the JE vaccine can also be considered for these areas.

Our study was not designed to collect vector species that transmit NMFs. However, in the ongoing malaria studies in the area, in the CDC light Trap, resting or larval breeding sites’ collections, specimens of *Aedes* and *Culex* vector species that transmit Dengue/Chikungunya and JE, respectively, are found (unpublished). The presence of these vector-borne diseases, even in winter months (December–February) (Figure 5), suggests the existence of perennial breeding sites of vectors. As Dengue and Chikungunya infections were found in the areas with dense forest cover (Figure 4a), without any travel history of the inhabitants, the possibility of new *Aedes* vectors transmitting these diseases and the involvement of different virus strains remains. In earlier NE India studies, DengueV-1 genotype-III and Asian-I of DengueV-2 were reported [13,22]. Reports of sylvatic Dengue [32] from some south-east Asian, African and Latin American countries [33,34,35] makes it worthwhile to initiate comprehensive vector and virus studies. It becomes imperative, more so, because the principal occupation of the villagers is *Jhum* cultivation with a lot of forest exposure. The majority of the positive and febrile patients were found using LLINs (Table S6). Hence, additional measures are required to prevent vector biting outside their homes. As only RDT tests were conducted for malaria, especially in the community surveillance, there can be some febrile cases positive for malaria, which are RDT negative but positive by a microscope, as it has been reported from this area [25]. It would also be worthwhile to look for the possible co-infections of NMVBDs in malaria-positive samples, as a subset of these samples showed it (unpublished). The diagnosis of NMVBDs along with malaria is very important in order to do proper case management. 

Many fevers can also be caused by water and foodborne pathogens, and UTI and respiratory infections, which are not studied here. Of 199 (77%) undiagnosed cases, in whom the aetiology remains unknown after screening for 6 specific aetiologic agents (including malaria), assessed in the present study, the majority of them showed symptoms of respiratory tract infections, among which an Upper Respiratory Tract Infection (URTI) includes 85% (*n* = 170) and a Lower Respiratory Tract Infection (LRTI) includes 13% (*n* = 25). The remaining 2% (*n* = 4) showed features of jaundice, vomiting, passing of dark urine and abdominal pain. Hence, it is crucial to conduct a study spanning the probable spectra of fever causing pathogens to investigate the aetiology of fevers in an area with a very high fever load. However, we would like to emphasise here, as mentioned before, there is a possibility that some more samples would have been detected for Dengue, Chikungunya, JE, and Scrub Typhus, had all the 260 samples been tested for all these diseases, irrespective of symptoms. Only a small subset of the samples from the community surveillance was tested for Leptospirosis, raising the possibility of increased detection of this infection in the full set. Furthermore, in this study, we have defined NMFs as fevers that are negative for malaria by RDT. It would be worth exploring if they were positive for malaria by microscopy or more sensitive methods, and contributing to fevers. There are several more sensitive diagnostic tools other than the gold standard light microscopy which are now being reported, some of which diagnose only *Plasmodium falciparum* while there are some for both *P. falciparum* and *P. vivax*, which are the predominant species in the study area. To mention a few, 18S nested PCR can detect 1–2 parasites of *P. falciparum* and 5–10 of *P. vivax* per microlitre [25,36]; micromagnetic resonance relaxometry [37] is an inexpensive instrument and, without any chemicals, it can detect about 10 parasites in a microlitre of blood; a multiplex single tube nested PCR that targets *Cytochrome Oxidase* III can diagnose all five human malaria parasites and is rapid with very high sensitivity [38]; an ultrasensitive surface enhancement Raman spectroscopy [39] and a rotating crystal magnet optical method detecting hemazoin are simple, rapid and diagnose both *P. falciparum* and *P. vivax* with high sensitivity and specificity [40]. Recently, a review was published on the advancements in malaria parasite diagnostics based on Omics [41]. Although the use of all these techniques at the rural PHC level would have many technical, operational and economical challenges, the introduction of sensitive and easy to operate techniques would increase the malaria positivity among the febrile cases.

One note of caution to be added here, in the highly malaria endemic areas, such as the current study area, a large load of submicroscopic, low-density malaria is found, where a sizeable proportion can be asymptomatic malaria [25], hence, in a febrile case, if low-density malaria is detected with highly sensitive detection kits, the cause of febrility can still be the other infections. Hence, studies should be undertaken to determine the comprehensive aetiologies using all existent diagnostic techniques, to explore all the surveillance possibilities at a rural hospital and community level, and to develop an integrated fever diagnosis and management system accordingly. 

Due to the ongoing pandemic, overlap in the clinical presentation and shunting of available resources and infection control in suspected COVID-19 patients can cause a delay in diagnosis and management of AFI [42]. As COVID-19 is demanding almost all the attention from the health system in terms of investment, resources and priority, and justifiably so in this health emergency, it is vital to ensure that other prevalent infectious diseases being responsible for high mortality and morbidity should not be ignored. Malaria is one such disease, along with NMVBDs. In this regard, the report on the occurrence of these vector-borne diseases and malaria in rural tribal malaria-endemic regions by community and health centre surveillance demands an integrated fever surveillance system for any kind of fever with some common symptoms in both the community and rural health centre levels. Its introduction and proper implementation can be helpful in the present as well as in the long run. 

## 5. Conclusions

This is the first study reporting the diagnosis of indigenous non-malarial vector-borne infections in malaria negative fever samples collected from the community fever surveillance in remote malaria-endemic tribal areas of India, indicative of local transmission for even Dengue and Chikungunya in deeply forested localities. Although these villages represent a very high fever load, malaria has been the only priority for fever surveillance in the villages and rural hospitals located in the malaria-endemic zone, where the presence of other vector-borne diseases are usually not attempted to diagnose. With the high positivity rates and many febrile cases not going beyond villages to seek treatment, studies to determine the actual prevalence and routine diagnostic surveillance, both at community and rural hospital level, even in the malaria-endemic region, become imperative. Findings from this study call for advances in routine diagnostic procedures and the development of a protocol that can accommodate currently, in practice, the rapid diagnosis of malaria and other vector-borne diseases, which are doable even in the resource-poor settings of rural hospitals and during community fever surveillance in order to prevent morbidity, mortality and outbreak situations. 

## Figures and Tables

**Table 1:** Total no. of fever and malaria cases reported in Ambassa PHC * during April 2019 to March 2020 period.

Ambassa PHC (Population ~61,000)	IPD *	OPD *	Total IPD + OPD	All SC * Cases	Total Ambassa PHC (IPD + OPD + SCs)
Fever cases	422	3100	3500	14,537	18,037
Malaria cases	155	3	158	431	589

* PHC = Primary Health Centre, IPD = Inpatient Department, OPD = Out Patient. Department, SC = Sub-Centre.

**Table 2:** Total no. of fever and malaria cases reported in Gurudhanpara and Shikaribari SC area during April 2019 to March 2020 period.

**Gurudhanpara SC (Population ~1900; Villages: Dhansinghpara, Bidyapara, Sambhuram, Sudhiram, Old Sudhiram, Khagendra, Tilakkumar)**						
	MPW *	ASHA *	**State and Project Village Health Volunteers and Project Staff**	**Health Camp**	**PHC IPD**	**Total**
**Fever**	489	564	525	65	33 **	1676
Malaria positive (by RDT *)	52	36	115	4	26**	233 (API ~122)
**Shikaribari SC (Population ~1800; Villages: Dankarai, Birendra, Lakhindra, Annaram, Satiram, Forest Village, Mahedra Debbarma, Rangachara, Sriratan, Mainayaram-Ananta 1, Mainayaram-Ananta 2)**						
**Fever**	479	115	279	7	28 *	908
**Malaria Positive (by RDT)**	34	6	51	0	11 **	102 (API ~57)
**Total Gurudhanpara + Shikaribari SC Fever Surveillance**						
**Fever**	968	679	804	72	61	2584
**Malaria Positive (by RDT)**	86	42	166	4	37	335

* RDT = Rapid Diagnostic Tests, MPW = Multipurpose Health Worker, ASHA = Accredited Social Health Activist. ** Cases referred to PHC from village or SC level surveillance are counted only once to avoid doubling of malaria cases.

**Table 3:** LLIN coverage and compliance pattern among tested and positive cases from villages. N denotes no. of persons, % in parenthesis represents LLINs use under different net categories.

Mosquito Net Type	JE * N, (%)	DEN * N, (%)	CHKN * N, (%)	ST * N, (%)	JE * + CHKN * N, (%)	DEN * + CHKN * N, (%)	DEN *+ ST * N, (%)	DEN * + JE * + CHKN * N, (%)	DEN * + CHKN * + ST * N, (%)
**New LLIN** **N, (%)**	3 (100)	12 (92)	6 (60)	5 (100)	3 (100)	3 (75)	1 (100)	3 (100)	1 (100)
**Old LLIN** **N, (%)**	0	1 (7.7)	3 (30)	0	0	1 (25)	0	0	0
**No LLIN**	0	0	1 (10)	0	0	0	0	0	0
**Period of sleep under a mosquito net**									
**Always**	3 (100)	12 (92.3)	7 (70)	5 (100)	3 (100)	4 (100)	1 (100)	3 (100)	1 (100)
**Sometimes**	0	1 (7.7)	2 (20)	0	0	0	0	0	0
**Never**	0	0	1 (10)	0	0	0	0	0	0

* DEN = Dengue, CHKN = Chikungunya, ST = Scrub Typhus, JE = Japanese Encephalitis, LLIN = Long-Lasting Insecticidal Nets.

## Data Availability

The data presented in this study are available on request from the corresponding author. The data are not publicly available due to ethical and privacy reasons.

## Supplementary Materials

The following are available online at https://www.mdpi.com/article/10.3390/diagnostics12020362/s1, Table S1. Eligibility criteria of Tripura IDSP and VRDL. The comprehensive eligibility criteria of Tripura IDSP and VRDL was used for testing different infections based on symptoms (IDSP = Integrated Disease Surveillance Program, VRDL = Viral Research Diagnostic Laboratory, ST = Scrub Typhus, JE = Japanese Encephalitis). Table S2. Total number of samples tested and positives for NMVBDs: IgMs of Dengue, Chikungunya, ST (Scrub Typhus), JE (Japanese Encephalitis) and Dengue NS1 antigen with different fever duration criteria. Table S3. Cluster analysis between the symptoms: The cluster analysis demonstrated a high degree of overlap between the symptoms for these three diseases. None of the four clusters corresponded to any single disease implying a lack of a defined set of symptoms for any of the 4 diseases. Table S4. Calculated true prevalence values of different infections as detected in village and hospital surveillance. For each disease, both single-disease and mixed-cases with other diseases were considered together in this calculation. There were few cases for Chikungunya and JE in PHC (one and three infections, respectively) and Dengue NS1 in both villages and PHC settings (two infections each) for the estimated PPV and NPV values to be meaningful; hence they were excluded. The specificity and sensitivity of the test kits used in this study are based on the manufacturers and the published results. Sensitivity and specificity values are considered as indicated in the kits (for Dengue and Chikungunya IgM kits), or the references cited there (for ST and NS1 kits), or mentioned for the kit elsewhere (JE IgM kit). Confidence limit calculations assume that sensitivity and specificity are known precisely. However, with variations observed in different studies [43,44,45] that used these kits, confidence limit calculations should be taken cautiously. (ST = Scrub Typhus, JE = Japanese Encephalitis). Table S5. Total number of samples falling in outside and within specified symptoms criteria. (JE = Japanese Encephalitis). Table S6. Demographic pattern and vector control practices: The demographic pattern and vector control practices among the febrile villagers tested at the community level, and all the positives for different diseases. The percentages of each count concerning the corresponding total are shown in the parenthesis. (JE = Japanese Encephalitis ST = Scrub Typhus). Figure S1. Percentage prevalence of the symptoms for each disease as reported by the patients. Mixed cases have not been considered for symptom calculations, hence sample numbers are low (only 3 for ST and Lepto). It shows that headache and body ache are the most common in all dis-eases. Myalgia is common in ~700% of cases of Dengue and CHKN, while arthralgia is common in 70% of Dengue and Lepto, and in ~50% of CHKN and JE cases. Chills were also reported in many cases of Dengue and JE. Irritability was found more commonly in JE. Rashes, typical for Dengue and Chikungunya, were not reported in these diseases, while they were reported in a small fraction of JE and ST cases. Retro orbital pain, another common symptom for Eschar, was not reported in any case. Reports of breathlessness were high from JE patients, while altered sensorium and a change of mental status was reported from very few patients, even in JE. A cough was reported in almost 70% of cases of all 4 diseases. (CHKN = Chikungunya, JE = Japanese Encephalitis, ST = Scrub Typhus, Leptospirosis).
