# Supplementary material for: Diagnosis of Indigenous Non-Malarial Vector-Borne Infections from Malaria Negative Samples from Community and Rural Hospital Surveillance in Dhalai District, Tripura, North-East India
Source: Diagnostics (Basel). 2022 Feb 1;12(2):362. doi: 10.3390/diagnostics12020362 (PMC8871021; doi:10.3390/diagnostics12020362)
Supplement: Supplementary file 1 [file diagnostics-12-00362-s001.zip › diagnostics-1529894-supplementary.pdf]

Supplementary Tables and Figures:

Table S1.

Syndromes	Associated Symptoms						
**Rash (Dengue, Chikungunya, ST)**	Fever	Macular	Papulae	Maculo-papular	Eschar	Pustule	
	Blister	Others					
**Encephalitis / Meningitis (JE, ST)**	Fever	Irritability	Increased Somnolence	New onset of seizures	Neck Rigidity	Altered Sensorium	Change in mental status
**Fever arthralgia (Dengue, Chikungunya, ST)**	Fever	Rigours	Headache	Chills	Malaise	Arthralgia	
	Myalgia	Hemorrhagic Manifestations		Retro-orbital Pain	Others		

Table S2.

	**Outside duration criteria IgM Test: Fever Duration (≤ 4 days)**	**IgM Test: Fever Duration (> 4 days)**
**Total Chikungunya Samples Tested**	85	149
**Chikungunya Positive**	3 (2 samples on 4th day and 1 on 3rd day)	9

**Mixed Infections**	**JE + Dengue + Chikungunya**	1 (4th day)	**JE + Chikungunya**	3
	**Dengue + Chikungunya**	3 (One each on day 2nd, 3rd and 4th day)	**Dengue + Chikungunya**	2
			**JE + Chikungunya + Dengue**	2
			**Dengue + Chikungunya + ST**	1
**Duration Dengue IgM**	**Outside duration criteria IgM Test: Fever Duration (≤ 4 days)**		**IgM Test: Fever Duration (> 4 days)**	
**Total Tested**	23		143	
**Dengue IgM Positives**	2 (on 3rd and 4th day)		13	
**Mixed Infections**	**Dengue+ Chikungunya**	3 (one each on 2nd, 3rd and 4th day)	**Dengue + Chikungunya + JE**	2
	**Dengue + ST**	1 (on 4th day)	**Dengue + Chikungunya**	1
	**JE + Dengue + Chikungunya**	1 (on 4th day)	**Dengue + Chikungunya + ST**	1
**Duration for Dengue NS1**	**Antigen (Dengue NS1) test: Fever Duration (≤ 4 days)**		**Outside duration criteria Antigen (Dengue NS1) test: Fever Duration (> 4 days)**	

<table>
<tr><td>Total Dengue NS1 Tested</td><td colspan="2">72</td><td colspan="2">26</td></tr>
<tr><td>Dengue NS1 positive</td><td colspan="2">2</td><td colspan="2">2 (On 5th day)</td></tr>
<tr><td>Duration for JE</td><td colspan="2">Outside duration criteria IgM Test: Fever Duration (≤ 4 days)</td><td colspan="2">IgM Test: Fever Duration (> 4 days)</td></tr>
<tr><td>Total JE Samples tested</td><td colspan="2">73</td><td colspan="2">119</td></tr>
<tr><td>JE positive</td><td colspan="2">0</td><td colspan="2">6</td></tr>
<tr><td rowspan="2">Mixed Infections</td><td rowspan="2">JE + Dengue+ Chikungunya</td><td rowspan="2">1 (on 4th day)</td><td>JE + Chikungunya</td><td>3</td></tr>
<tr><td>JE + Chikungunya + Dengue</td><td>2</td></tr>
<tr><td>Duration ST IgM</td><td colspan="2">Outside duration criteria IgM Test: Fever Duration (<7 days)</td><td colspan="2">IgM Test: Fever Duration (>7 days)</td></tr>
<tr><td>Total ST Samples Tested</td><td colspan="2">137</td><td colspan="2">72</td></tr>
<tr><td>ST Positive</td><td colspan="2">3 (2 on 4th Day & 1 on 6th day)</td><td colspan="2" rowspan="3">6</td></tr>
<tr><td rowspan="2">Mixed Infections</td><td>Dengue + ST</td><td>1 (4th Day)</td></tr>
<tr><td>Dengue + Chikungunya + ST</td><td>1 (6th Day)</td></tr>
</table>

Table S3:

	Scrub Typhus	Dengue	Chikungunya	JE
**True Prevalence (Blaker CL)**	0.043	0.134	0.0842	0.0457
**True Prevalence (Blaker CL) Lower 95 % CL**	0.015	0.0851	0.0499	0.0173
**True Prevalence (Blaker CL) Upper 95 % CL**	0.090	0.2001	0.1324	0.0925
**Positive predictive value**	0.69	0.8802	0.8137	0.6946
**Negative predictive value**	0.992	0.9922	0.9953	0.9976
**Likelihood Ratio +ve**	49.529	47.5	47.5	47.5
**Likelihood Ratio -ve**	0.160	0.051	0.051	0.051

Table S4

k Mode Clusters	JE	Chikungunya	Dengue	Scrub Typhus
1	3	4	8	2
2	1	2	2	4
3	0	2	5	0
4	2	4	4	3

Table S5:

Outside specified symptoms criteria	JE	Dengue	Chikungunya
**Samples Tested**	118	16	19
**Positives**	3	0	0
**Within specified symptoms criteria**	**JE**	**Dengue**	**Chikungunya**
**Samples tested**	74	220	215
**Positives**	3	18	12

Table S6:

Houses with	Diseases									
	**JE**	**Dengue**	**Chikungunya**	**ST**	**JE + Chikungunya**	**Dengue + Chikungunya**	**Dengue + ST**	**Dengue+ JE +Chikungunya**	**Dengue+ Chikungunya+ ST**	**Total febrile cases tested at the community level**
	**Roof Type N=Number of houses (%)**									
**Bamboo**	0	2 (15.4)	1 (100)	0	0	0	0	0	0	6 (3.3)

**Thatch**	0	2 (15.4)	5 (50)	1 (20)	0	1 (25)	0	0	0	36 (19.7)
**Tin**	3 (100)	9 (69.2)	4 (40)	4 (80)	3 (100)	3 (75)	1 (100)	3 (100)	1 (100)	141 (77.1)
	**Wall type N=Number of houses (%)**									
**Bamboo**	2 (66.7)	4 (30.8)	6 (60)	1 (20)	2 (66.7)	1 (25)	0	3 (100)	0	67 (35.8)
**Cement**	0	3 (23.1)	1 (10)	0	1 (33.3)	0	1 (100)	0	0	25 (13.4)
**Mixed**	1 (33.3)	0	0	1 (20)	0	0	0	0	0	11 (5.9)
**Mud**	0	4 (30.8)	2 (20)	3 (60)	0	2 (50)	0	0	1 (100)	71 (37.9)
**Mud Bamboo**	0	0	1 (10)	0	0	1 (25)	0	0	0	3 (1.6)
**Tong Ghar**	0	2 (15.4)	0	0	0	0	0	0	0	10 (5.4)
	**Livestock Shelter (N, %)**									

**Yes**	0	4 (30.8)	1 (10)	1 (20)	1 (33.3)	2 (50)	0	0	0	48 (26.2)
**No**	3 (100)	9 (69.2)	9 (90)	4 (80)	2 (66.7)	2 (50)	0	3 (100)	1 (100)	135 (73.8)
	**Occupation (N, %)**									
**Daily Wage Labourer**	0	0	0	0	2 (66.7)	0	0	0	0	4 (2.2)
***Jhum* Cultivators**	2 (66.7)	5 (38.5)	4 (40)	3 (60)	1 (33.3)	0	1 (100)	2 (66.7)	0	89 (48.4)
**Others**	0	2 (15.4)	2 (20)	0	0	1 (25)	0	0	0	20 (10.9)
**Service**	0	1 (7.7)	0	0	0	0	0	0	0	3 (1.6)
**Student**	1 (33.3)	5 (38.5)	4 (40)	2 (40)	0	3 (75)	0	1 (33.3)	1 (100)	68 (36.9)

Figure S1:

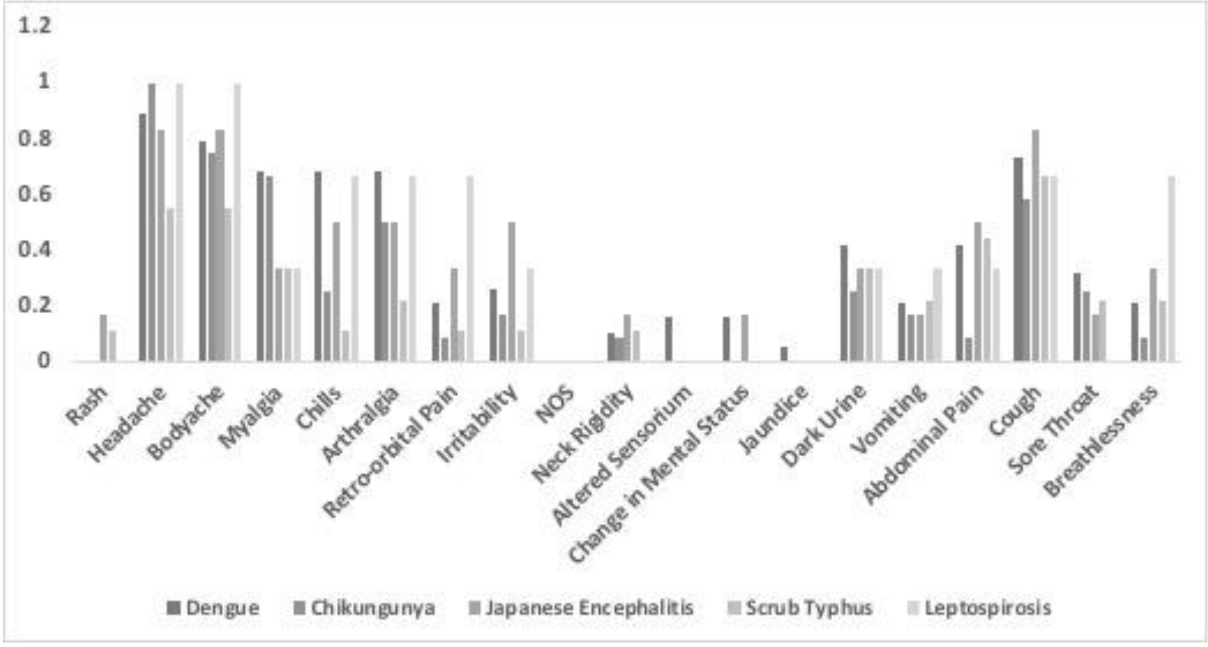

Supplementary Materials: The following are available online at https://www.mdpi.com/article/10.3390/diagnostics12020362/s1, Table S1. Eligibility criteria of Tripura IDSP and VRDL. The comprehensive eligibility criteria of Tripura IDSP and VRDL was used for testing different infections based on symptoms (IDSP = Integrated Disease Surveillance Program, VRDL = Viral Research Diagnostic Laboratory, ST = Scrub Typhus, JE = Japanese Encephalitis). Table S2. Total number of samples tested and positives for NMVBDs: IgMs of Dengue, Chikungunya, ST (Scrub Typhus), JE (Japanese Encephalitis) and Dengue NS1 antigen with different fever duration criteria. Table S3. Cluster analysis between the symptoms: The cluster analysis demonstrated a high degree of overlap between the symptoms for these three diseases. None of the four clusters corresponded to any single disease implying a lack of a defined set of symptoms for any of the 4 diseases. Table S4. Calculated true prevalence values of different infections as detected in village and hospital surveillance. For each disease, both single-disease and mixed-cases with other diseases were considered together in this calculation. There were few cases for Chikungunya and JE in PHC (one and three infections, respectively) and Dengue NS1 in both villages and PHC settings (two infections each) for the estimated PPV and NPV values to be meaningful; hence they were excluded. The specificity and sensitivity of the test kits used in this study are based on the manufacturers and the published results. Sensitivity and specificity values are considered as indicated in the kits (for Dengue and Chikungunya IgM kits), or the references cited there (for ST and NS1 kits), or mentioned for the kit elsewhere (JE IgM kit). Confidence limit calculations assume that sensitivity and specificity are known precisely. However, with variations observed in different studies [43–45] that used these kits, confidence limit calculations should be taken cautiously. (ST = Scrub Typhus, JE = Japanese Encephalitis). Table S5. Total number of samples falling in outside and within specified symptoms criteria. (JE = Japanese Encephalitis). Table S6.

Demographic pattern and vector control practices: The demographic pattern and vector control practices among the febrile villagers tested at the community level, and all the positives for different diseases. The percentages of each count concerning the corresponding total are shown in the parenthesis. (JE = Japanese Encephalitis ST = Scrub Typhus). Figure S1. Percentage prevalence of the symptoms for each disease as reported by the patients. Mixed cases have not been considered for symptom calculations, hence sample numbers are low (only 3 for ST and Lepto). It shows that headache and body ache are the most common in all dis-eases. Myalgia is common in ~700% of cases of Dengue and CHKN, while arthralgia is common in 70% of Dengue and Lepto, and in ~50% of CHKN and JE cases. Chills were also reported in many cases of Dengue and JE. Irritability was found more commonly in JE. Rashes, typical for Dengue and Chikungunya, were not reported in these diseases, while they were reported in a small fraction of JE and ST cases. Retro orbital pain, another common symptom for Eschar, was not reported in any case. Reports of breathlessness were high from JE patients, while altered sensorium and a change of mental status was reported from very few patients, even in JE. A cough was reported in almost 70% of cases of all 4 diseases. (CHKN = Chikungunya, JE = Japanese Encephalitis, ST = Scrub Typhus, Leptospirosis).
